# Inhibition of Mammalian Target of Rapamycin Complex 1 (mTORC1) Downregulates *ELOVL1* Gene Expression and Fatty Acid Synthesis in Goat Fetal Fibroblasts

**DOI:** 10.3390/ijms160716440

**Published:** 2015-07-20

**Authors:** Weipeng Wang, Qiburi He, Zhixin Guo, Limin Yang, Lili Bao, Wenlei Bao, Xu Zheng, Yanfeng Wang, Zhigang Wang

**Affiliations:** 1 College of Life Sciences, Inner Mongolia University, Hohhot 010021, China; E-Mails: quintas@live.cn (W.W.); qbr2010@126.com (Q.H.); GZX770461760@163.com (Z.G.); yangliminlh@163.com (L.Y.); baolili1203@126.com (L.B.); bwlnhm@sina.com (W.B.); dong2186@163.com (X.Z.); 2 College of Basic Medical Science, Inner Mongolia Medical University, Hohhot 010110, China; 3 Hulunbeir Municipal People’s Hospital, Hailar 021008, China

**Keywords:** cashmere goat, ELOVL1, expression pattern

## Abstract

Elongation of very-long-chain fatty acids 1 (*ELOVL1*) is a ubiquitously expressed gene that belongs to the ELOVL family and regulates the synthesis of very-long-chain fatty acids (VLCFAs) and sphingolipids, from yeast to mammals. Mammalian target of rapamycin complex 1 (mTORC1) is a central regulator of cell metabolism and is associated with fatty acids synthesis. In this study, we cloned the cDNA that encodes Cashmere goat (*Capra hircus*) ELOVL1 (GenBank Accession number KF549985) and investigated its expression in 10 tissues. *ELOVL1* cDNA was 840 bp, encoding a deduced protein of 279 amino acids, and *ELOVL1* mRNA was expressed in a wide range of tissues. Inhibition of mTORC1 by rapamycin decreased *ELOVL1* expression and fatty acids synthesis in Cashmere goat fetal fibroblasts. These data show that ELOVL1 expression is regulated by mTORC1 and that mTORC1 has significant function in fatty acids synthesis in Cashmere goat.

## 1. Introduction

Fatty acids (FAs) are components of most cellular lipids, such as glycerolipids, sphingolipids, and cholesterol esters, that are classified by their carbon (C) chain length, including long-chain FAs (LCFAs) with carbon (C) chain lengths of 11/12–20, and of which C16 and C18 LCFAs are the most abundant FA species in mammalian cells. Very long-chain FAs (VLCFAs) are defined as C ≥ 22 and less abundant than LCFAs [1,2].

C22 and C24 VLCFAs are ubiquitous throughout the body, and VLCFAs with C ≥ 26 are often subgrouped into ultra-long-chain FAs (ULCFAs) and are expressed in specific tissues, including skin, retina, meibomian gland, testis, and brain [2]. VLCFAs are classified as saturated FAs (SFAs), monounsaturated FAs (MUFAs) and polyunsaturated FAs (PUFAs), each of which has distinct functions and properties [3]. VLCFAs are pleiotropic, important for cellular and metabolic functions, including intracellular signaling and protein transport, and with several disorders.

In mammals, fatty acids that comprise up to 16 carbons (palmitic acid) in length are synthesized and elongated into long-chain fatty acids that contain 18 carbon atoms or VLCFAs. The formation of VLCFA is effected primarily by endoplasmic reticulum (ER) membrane-embedded enzymes [1].

FA elongation occurs by cycling through a four-step process (condensation, reduction, dehydration, and reduction) [1,4]. In the elongation of very-long-chain fatty acids, FA elongase catalyzes the rate-limiting condensation step during the sequential VLCFA elongation cycle. A recently report investigated the kinetic characteristic of ELOVL1 (Elongation of very-long-chain fatty acids 1) and demonstrated that ELOVL1 is indeed the rate-limiting enzyme and ELOVL1 was the most potent elongase for C22:0, C24:0 and C26:0 [3,5]. Seven elongases (ELOVL1–7) have been identified and characterized in mammals, each of which has its specific substrate and function [6]. However, their exact activities in VLCFA elongation have not been determined.

*ELOVL1* is a ubiquitously expressed gene, the product of which has been suggested to be involved in the production of C24 sphingolipids in yeast and mammals, and is implicated in the synthesis of saturated and monounsaturated C24 sphingolipids [3,7]. Mammalian cells contain high amounts of sphingolipids with C16:0, C24:0 or C24:1 FAs, and ELOVL1 is the chief FA elongase that mediates the production of C24 sphingolipids [1,3]. Sphingolipids abound in the plasma membrane and are important in maintaining membrane functions.

Little is known about the regulation of *ELOVL1*, although it is highly expressed in tissues [4]. Ohno *et al.* [3] reported that ELOVL1 activity depends on ceramide synthase 2 (CerS2) expressions, and v*ice versa* [8]. ELOVL1 may form complexes with other related proteins to play elongase function. A recent study demonstrated that oleic and erucic acids could inhibit ELOVL1 and decrease the level of sphingomyelin with a saturated very-long-chain fatty acid [9] in humans. However, the regulatory mechanisms of *ELOVL1* expression remain unknown.

Mammalian target of rapamycin (mTOR), which was renamed mechanistic target of rapamycin (MTOR) by the Human Genome Organisation (HUGO) Gene Nomenclature Committee (HGNC), is a central regulator of cell growth and metabolism and is sufficient to induce specific metabolic processes, including *de novo* lipid biosynthesis [10,11,12]. A gene set enrichment analysis (GSEA) of a large set of genes by microarray showed that mTORC1 induces ELOVL1 expression in tuberous sclerosis 2 gene knockout (Tsc2^−/−^) cells [10], suggested that *ELOVL1* is a downstream regulatory target of mTORC1.

To study ELOVL1 function and its relationship with mTORC1 during lipid synthesis in Cashmere goat cells, we cloned *ELOVL1* cDNA from Inner Mongolia Cashmere goat (*Capra hircus*), analyzed its expression in 10 tissues, and predicted the function of goat ELOVL1 by bioinformatics analysis. Furthermore, we examined the effects of rapamycin, a specific inhibitor of mTORC1, on *ELOVL1* expression and fatty acid synthesis in Cashmere goat fetal fibroblasts. Through a combination of molecular genetic, metabolic, and bioinformatic analyses, we find that *ELOVL1* expression is regulated by mTORC1, and mTORC1 has significant function in fatty acid synthesis in Cashmere goat cells.

## 2. Results

### 2.1. cDNA Cloning and Sequence Analysis

**Figure 1:**
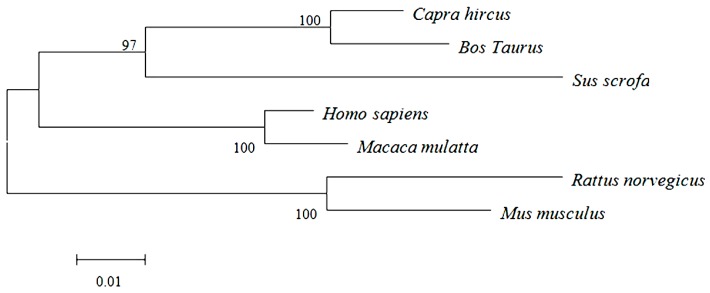
Phylogenetic tree of *ELOVL1*. Goat *ELOVL1* was aligned with other *ELOVL1* homologs, and a phylogenetic tree was constructed by neighbor-joining method using MEGA4.1. The species and GenBank accession numbers are *Capra hircus* (KF549985), *Bos Taurus* (NM001034703.2), *Sus scrofa* (NM001167647.2), *Homo sapiens* (NM022821.3), *Macaca mulatta* (NM001261559.1), *Rattus norvegicus* (NM001044275.1), and *Mus musculu*s (NM001039176. 2). The scale of evolutionary distance is 0.01.

*ELOVL1* cDNA (GenBank Accession number KF549985) from Inner Mongolia Cashmere goat comprises an open reading frame (ORF) of 840 bp. The sequence is 87% identical to rat *ELOVL1* and 88%, 91%, 91%, 92% and 97% identical to mouse, pig, monkey, human and bovine *ELOVL1*, respectively. To determine the phylogenetic relationships between *ELOVL1* of various species, the nucleotide sequence was aligned with those of other homologs, and a phylogenetic tree was constructed, based on these alignments (Figure 1).

### 2.2. Primary and Secondary Structure of Cashmere Goat ELOVL1 Protein

**Figure 2:**
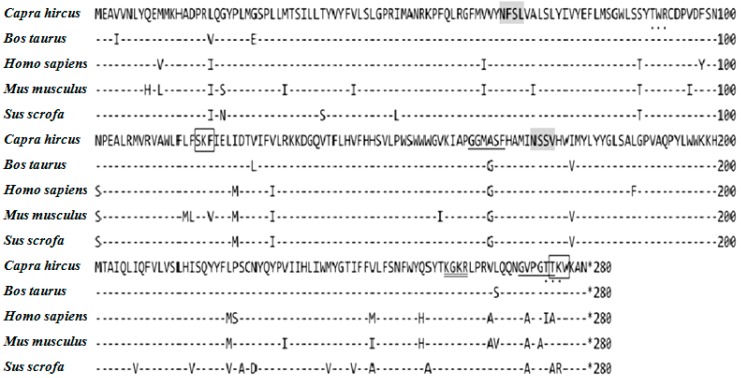
Deduced amino acid sequence and the active sites of ELOVL1. Alignment of amino acid sequences of *capra hircus* (KF549985), *bos taurus* (NM001034703.2), *sus scrofa* (NM001167647.2), *homo sapiens* (NM022821.3), and *mus musculus* ELOVL1 (NM001039176. 2). Predicted *N*-glycolsylation sites are shaded gray. Protein kinase C phosphorylation sites are indicated as dots. *N*-myristoylation sites are underlined. Amidation sites are marked by double underlines. Microbody C-terminal targeting signals are boxed. All sites were determined using Psite (Available online: http://www.softberry.com).

The deduced primary structure of Inner Mongolia Cashmere goat ELOVL1 consists of 279 amino acids, and the predicted molecular weight is 32,651 Da for the unmodified protein; the estimated isoelectric point (p*I*) is 5.05. There are two *N*-glycosylation sites, two protein kinase C phosphorylation sites, two *N*-myristoylation sites, one amidation site and two C-terminal microbody targeting signals (Figure 2). ELOVL1 is an integral membrane protein that is embedded in the endoplasmic reticulum membrane.

**Figure 3:**
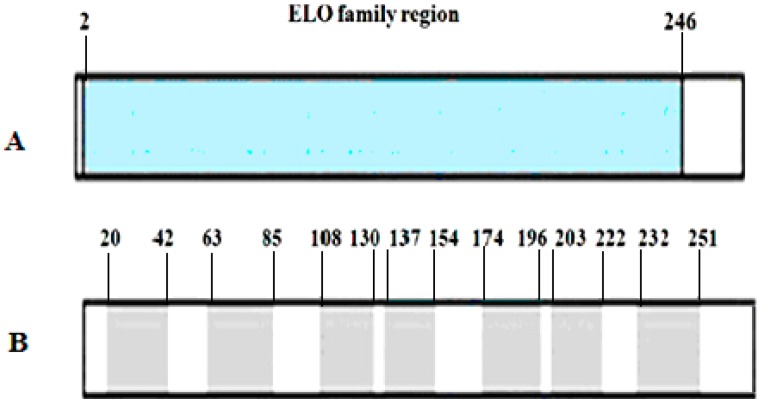
Predicted domains of goat ELOVL1. (**A**) ELO family region predicted by Pfam from amino acid residues 2–246; (**B**) Seven transmembrane regions from amino acid residues 20 to 42, 63 to 85, 108 to 130, 137 to 154, 174 to 196, 203 to 222, and 232 to 251.

Our sequence search results from Pfam database suggested that ELOVL1 contains an ELO family region (residues 2–246) (Figure 3A); by SMART search, there are seven transmembrane regions (spanning residues 20–42, 63–85, 108–130, 137–154, 174–196, 203–222, and 232–251) (Figure 3B).

### 2.3. Tissue Distribution of Cashmere Goat ELOVL1 mRNA

**Figure 4:**
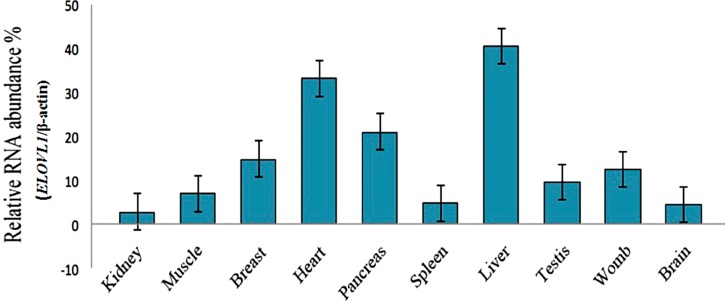
Tissue distribution of *ELOVL1* transcripts. *ELOVL1* mRNA was analyzed by quantitative real-time RT-PCR with the SYBR^®^ Premix ExTaq™ (Perfect Real Time, TaKaRa Co., Ltd., Dalian, China) system. Relative RNA abundance % is the relative fold of β-actin. mRNA levels were highest in liver compared with kidney, muscle, breast, heart, pancreas, spleen, testis, womb, and brain tissue.

The relative abundance of *ELOVL1* mRNA was measured in Cashmere goat skin, brain, heart, muscle, lung, liver, pancreas, spleen, kidney, testis, womb, and mammary gland by quantitative real-time PCR. *ELOVL1* mRNA expressed in all tissues, with the liver and heart having greater relative abundance (Figure 4).

### 2.4. Rapamycin Down-Regulates the Transcription of ELOVL1 in GFb Cells

**Figure 5:**
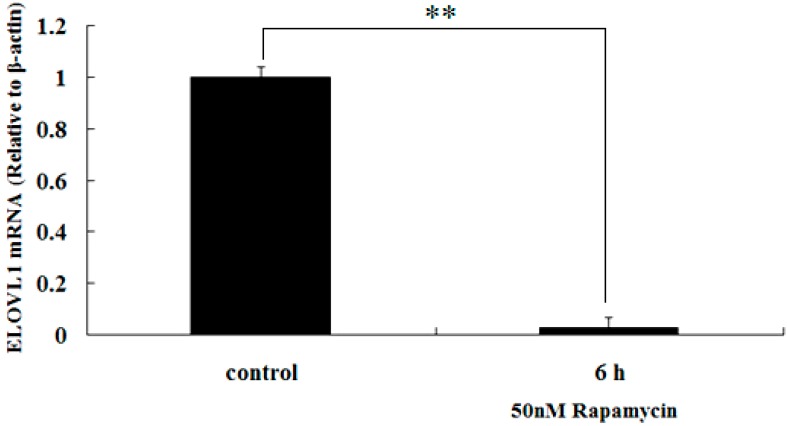
Rapamycin inhibits the relative abundance of *ELOVL1* mRNA in GFb cells. Cells were treated with 50 nM rapamycin for 6 h, and *ELOVL1* mRNA was detected by real-time qPCR. The relative abundance of *ELOV1* mRNA in the treated GFb cells was significantly inhibited (** *p* < 0.01).

To determine whether mTORC1 regulates the transcription of *ELOVL1* in GFb cells, we studied the effects of rapamycin on the relative abundance of *ELOVL1* mRNA in GFb cells. Cells were treated with 50 nM rapamycin for 6 h, and *ELOVL1* mRNA was detected by real-time qPCR. The results showed that rapamycin inhibited the relative abundance of *ELOV1* mRNA in the treated GFb cells (Figure 5), suggesting that transcription of *ELOV1* was significantly down regulated (*p* < 0.01).

### 2.5. Rapamycin Attenuates ELOVL1 Expression and Fatty Acid Synthesis in GFb Cells

**Figure 6:**
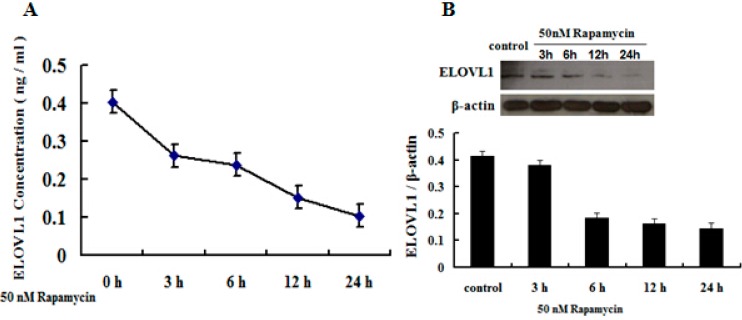
Rapamycin induces a time-dependent decrease in the expression of ELOVL1. Cells were treated with 50 nM rapamycin for 3, 6, 12 and 24 h, and ELOVL1 in cells was detected by ELISA (**A**) and Western blot (**B**).

To determine whether mTORC1 regulates the expression of *ELOVL1* and fatty acid synthesis in GFb cells, we studied the effects of rapamycin on different levels. Cells were treated with 50 nM rapamycin for 3, 6, 12 and 24 h, and ELOVL1 was detected by ELISA and Western blot. Fatty acids were extracted from cells that were treated with 50 nM rapamycin for 6 h and assayed by GC-MS. Rapamycin induced a time-dependent decrease in ELOVL1 expression (Figure 6), and the levels of certain type fatty acids declined due to rapamycin (Table 1), suggesting that ELOVL1 expression is regulated by mTORC1 and mTORC1 has function in fatty acid synthesis in Cashmere goat cells.

### 2.6. Rapamycin Inhibits p70S6K (Thr 389) Expression in a Time- and Dose-Dependent Manner

**Figure 7:**
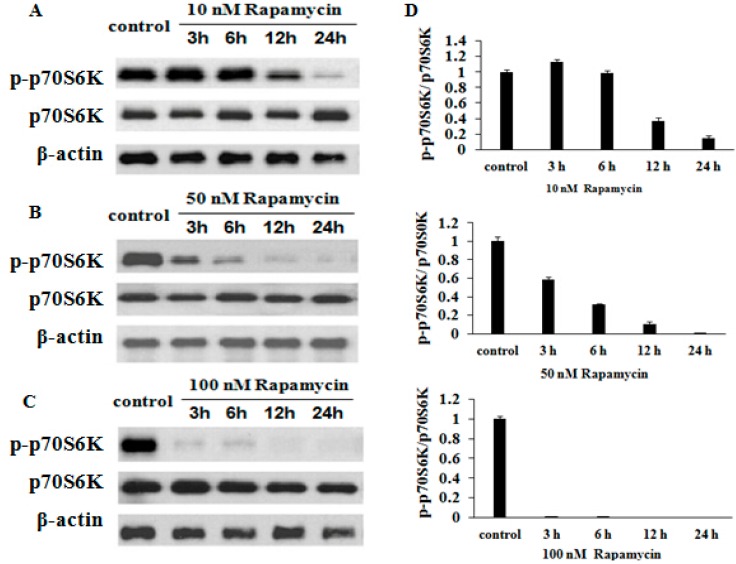
Rapamycin downregulates p70S6K (Thr 389) in GFb cells. Cells were treated with 10, 50 and 100 nM rapamycin for 3, 6, 12 and 24 h, and the expression of p70S6K, and p70S6K (Thr 389) were measured by Western blot. (**A**) Treatment with 10 nM. Phosphorylation of p70S6K (Thr 389) decreased after treatment for 12 h; (**B**) Treatment with 50 nM. Phosphorylation of p70S6K (Thr 389) was suppressed after 3 h; (**C**) Treatment with 100 nM. Phosphorylation of p70S6K (Thr 389) fell significantly after treatment for 3 h; (**D**) The resolved bands were quantified using Gel-Pro Analyzer 4.0 (Media Cybernetics, Inc., Rockville, MD, USA).

To determine the mechanism of rapamycin inhibition in GFb cells, we measured the expression of p70S6K and phosphorylation of p70S6K (Thr 389) by Western blot. On treatment of cells with 10, 50 and 100 nM rapamycin for 3, 6, 12 and 24 h, phospho-p70S6K (Thr 389) were downregulated with increasing treatment doses and times (Figure 7A–D), indicating that rapamycin inhibits the expression of these proteins dose- and time-dependently.

**Table 1:** Content of fatty acids in control and rapamycin-treated cells.

Fatty Acid	Control (mg/kg)	Treatment (mg/kg)
Undecanedioic acid (11:0)	0.12 ± 0.004	0.11 ± 0.009
Tridecanoic acid (13:0)	0.04 ± 0.008	0.04 ± 0.008
Myristic acid (14:0)	4.62 ± 0.48	4.21 ± 0.29
Pentadecanoic acid (15:0)	0.44 ± 0.01	0.41 ± 0.03
Palmitic acid (16:0)	606.42 ± 22.50	565.10 ± 32.51
Margaric acid (17:0)	4.07 ± 0.02	3.84 ± 0.35
Stearic acid (18:0)	721.06 ± 36.82	664.16 ± 30.31
Oleic acid (18:1)	7.16 ± 1.97	6.45 ± 0.62
Linoleic acid (18:2)	1.36 ± 0.24	1.37 ± 0.28
Arachidic acid (20:0)	3.72 ± 0.14	3.34 ± 0.27
Heneicosanic acid (21:0)	0.58 ± 0.04	0.55 ± 0.05
Behenic acid (22:0)	0.22 ± 0.02	0.19 ± 0.03
Erucic acid (22:1)	2.36 ± 0.15	2.13 ± 0.25
Tricosanoic acid (23:0)	0.03 ± 0.01	0.01 ± 0.004

## 3. Discussion

Long-chain FAs are synthesized by FA synthase (FAS) and converted into VLCFAs by ER membrane-bound enzymes [4,13], such as ELOVL1. The genes that encode these enzymes have been characterized extensively in yeast, including ELO2/FEN1, ELO3/SUR4, YBR159w, PHS1, TSC13, and Phs1 [13,14]. FA biosynthesis pathways have also been studied frequently in abyssal fish species due to the significant effects of docosahexaenoic acid (DHA, 22:6n−3) and eicosapentaenoic acid (EPA, 20:5n−3) on the human brain [15]. There is substantial evidence on ELOVLs encode enzymes that mediate the biosynthesis of PUFA in fish.

However, lipid synthesis activities in yeast and fish do not always correspond to such processes in mammals. With regard to lipid synthesis, mammals have seven condensation enzymes (ELOVL1–7) and four 3-hydroxyacyl-CoA dehydratases (HACD1–4). Nevertheless, whether mammalian enzymes also form elongase complexes or whether individual HACD protein interact with specific ELOVL1 nor the interaction within ELOVLs family remains unknown [13]. Researchers have focused on the mammalian ELOVL family to facilitate the study of these complexes and interaction between the enzymes [3,7,10], the exact mechanism of which remains unknown.

ELOVL1 was discovered as a mammalian homolog to ELO3/SUR4 in yeast [6,16], which mediates tissue-specific biosynthesis of VLCFAs and sphingolipids, and it is believed to be a functional ortholog of yeast ELO3 in human and mouse [6,17]. In this study, we cloned *ELOVL1* from Cashmere goat and observed that goat *ELOVL1* also encodes 279 amino acids and shares 88% and 92% identity with the mouse and human homologs. The deduced amino acid sequence of goat ELOVL1 contains an ELO family region, a homolog of yeast ELO.

ELO belongs to the GNS1/SUR4 family, which mediates long-chain fatty acid elongation and the generation of 26-carbon precursors for ceramide and sphingolipid synthesis [16]. Moreover, we detected *ELOVL1* in 10 goat tissues by real-time PCR, similar to the expression pattern in human [3]. Thus, we hypothesize that Cashmere goat ELOVL1 is an ortholog of yeast ELO. *ELOVL1* is evolutionarily conserved.

A previous report has demonstrated that mTORC1 promotes lipid biogenesis by regulating the expression of lipogenic genes [10], the products of which are related to sterol regulatory element-binding proteins (SREBPs) and peroxisome proliferator-activated receptors (PPARs), which are two important types of transcription factors related to *de novo* lipid biosynthesis [18,19,20]. mTORC1 controls peripheral nervous system (PNS) myelination along the mTORC1-RXRγ-SREBP-lipid biosynthesis axis in Schwann cells [21]. The activation of SREBP1/2 is mediated by S6K1 [10]. Treatment with leucine has recently been shown to upregulate mTOR, S6K1, and SREBP-1c in dairy cow mammary epithelial cells [22], suggested that they are coexpressed during milk synthesis.

mTORC1 affects adipogenesis by modulating the expression and activity of PPARγ, which govern the expression of genes that are required for fatty acid synthesis, uptake, and esterification [19]. Furthermore, the expression of *ELOVL* genes is associated with SREBPs and PPARs [9,23,24,25] and the expression of *ELOVL1* and *ELOVL5* was induced by mTORC1, and that mTORC1 activates SREBP1 through S6K1 in MEFs [10]. Thus, we hypothesize that mTORC1 regulates the expression of lipid biogenesis-related genes through the transcription factors SREBP1 and PPARγ.

In the present study, ELOVL1 expression is downregulated about 50%, as analyzed by ELISA, and 60%, as analyzed by Western blot, after six hours of 50 nM rapamycin treatment (Figure 6). In a previous publication, ELOVL1 protein expression was reduced by 80% at six days using siRNA to knockdown ELOVL1 in X-linked adrenoleukodystrophy (X-ALD) fibroblasts [17]. These two results seem inconsistent and rapamycin shows rapid effect. In fact, the rapid inhibition of rapamycin has been found in other cells. Kuo *et al.* (2011) reported that c-Myc and cyclin D3 were reduced after 4 h treatment with 20 nM RAD001, which is a specific inhibitor of mTORC1, in lymphoma cells Pfeiffer and MC116. However, the p27 expression was not affected [26]. In a recently publication, Li *et al.* [27], used 20 nM rapamycin treated the human breast cancer cell lines MDA-MB-231 for 2 h, the HIF-1α expression was heavily reduced under both normoxic and hypoxic conditions. Furthermore, Chen *et al.* [28] reported that rapamycin (20 nmol/L, 12 h) down regulated the expression of fatty acid synthase (FASN), which is the enzyme for the cellular synthesis of palmitate, whereas the expression of 4EBP-1 and p70S6K did not change. Moreover, transcription of *ELOV1* was significantly down regulated in our present data (Figure 5). So, rapamycin may inhibit some gene transcription specifically in a short time and the exact mechanism is not well known.

In this study, we examined *ELOVL1* expression and inhibition of mTORC1 by rapamycin in Cashmere goat fetal fibroblasts, and found that rapamycin downregulated *ELOVL1* expression and fatty acid synthesis. These data indicate that *ELOVL1* expression is regulated by mTORC1 and that mTORC1 has a significant effect on fatty acids synthesis in Cashmere goat.

## 4. Experimental Section

### 4.1. Animal and Tissue Collection

Tissue samples were dissected from 3 male and 3 female adult Inner Mongolia Cashmere goats that bred on a natural diet in Inner Mongolia, China. Skin, brain, heart, muscle, lung, liver, pancreas, spleen, kidney, testis, womb, and mammary gland were sampled after the animals were sacrificed in a commercial abattoir in autumn. Tissue samples were flash-frozen in liquid nitrogen immediately after harvest and then stored at −80 °C.

### 4.2. Cell Culture Conditions

Inner Mongolia Cashmere goat fetal fibroblasts (GFb cells) were maintained as monolayer cultures in DMEM/F12 (D-MEM/F-12, Gibco, Paisley, PA49RF, Scotland, UK), supplemented with 10% fetal bovine serum (FBS) (Hyclone Laboratories, Inc., Logan, UT, USA), 100 U/mL penicillin G, and 100 mg/mL streptomycin (Sigma-Aldrich, Inc., St. Louis, MO, USA). Cell cultures were maintained and incubated at 37 °C in humidified air with 5% CO_2_. Morphology was examined by light microscopy.

### 4.3. Total RNA Extraction and Full-Length cDNA Isolation

Total RNAs were extracted from Inner Mongolia GFbs using RNAzol (RNAiso Plus, Takara Co., Ltd., Dalian, China). The concentration of total RNA was measured with Nanodrop ND-1000 spectrophotometer (Scientific, Waltham, MA, USA), and the integrity was examined on an agarose gel. Reverse-transcription reactions were performed using 1 μg total RNA in a 20-μL setup with the M-MLV 1st Strand cDNA Synthesis kit (Takara Co., Ltd., Dalian, China) per the manufacturer’s instructions with oligo (dT)_12–18_ primer.

### 4.4. Cloning and Sequencing of ELOVL1 cDNA

A pair of specific degenerate primers were designed, based on the *Bos taurus* ELOVL1 sequence (GenBank Accession number NM_001034703.2), to amplify Inner Mongolia Cashmere goat *ELOVL1* cDNA. The CDS fragment of *ELOVL1* was amplified by PCR for 35 cycles with full-length cDNA as template at the appropriate annealing temperature for the following primer pair: forward: 5′-GCGAATTCATGGAGGCTA/GTTGTGAACC/TTG-3′ and reverse: 5′-GCGGATCCGC/TTTCTCAGTTGGCCTTGACC/T-3′. (*Eco*R I and *Bam*H I sites are underlined).

PCR products were electrophoresed, and photographs were taken on a UV transilluminator (UVItec, London, UK). The amplicons were purified, cloned into pMD19-T (Takara Co., Ltd., Dalian, China), and sequenced on an ABI PRISM 377XL DNA Sequencer (Applied Biosystems, Inc., Foster City, CA, USA). The predicted fragment length was 840 bp.

### 4.5. Tissue Distribution Analysis and Relative Abundance in GFb Cells of ELOVL1 mRNA by Real-Time q-PCR

Real-time quantitative polymerase chain reaction (q-PCR) was performed to determine the abundance of *ELOVL1* mRNA in a variety of tissues from adult Cashmere goat, which includes skin, brain, heart, muscle, lung, liver, pancreas, spleen, kidney, testis, womb, and mammary gland. *ELOVL1* was amplified with the following primers: forward 5′-GGAAGAAAGACGGACAGGTGAC-3′ and reverse 5′-CCAAGGGCAGACAATCCATAG-3′. β-actin was used as an internal control to normalize RNA loading, and the primers were designed, based on the *Capra hircus* β-actin sequence (GenBank Accession number AF481159.1). β-actin was amplified with the following primers: forward 5′-CCAAGGGCAGACAATCCATAG-3′ and reverse 5′-CGTCCCCAGAGTCCATGACAATG-3′.

Quantitative real-time PCR was performed on a Rotor Gene Q (Qiagen, Hilden, Germany) using SYBR^®^ Premix Ex Taq™ (Perfect Real Time). One microliter of cDNA was amplified in a 25-μL mixture that contained 10 mM forward primer (0.5 mL), 10 mM reverse primer (0.5 μL), 2 SYBR^®^ Premix Ex Taq™ (12.5 μL), and nuclease-free water (10.5 μL). The program comprised an initial denaturation step at 95 °C for 5 min; 40 cycles at 95 °C for 5 s, 54 °C for 30 s, and 72 °C for 20 s; 72 °C for 10 min; and a final melting step. Three technical replicates were run.

Delta CT (ΔCT) values were calculated to determine tissue-specific expression. The real-time PCR results were analyzed by one-way analysis of variance (ANOVA) to compare expression between tissues.

The relative abundance of *ELOVL1* mRNA in cells of treated group and control was amplified with following primers: forward 5′-GCTCAGCCCTACCTTTG-3′ and reverse 5′-CCTGGAATCCCGTTTTG-3′.

### 4.6. Bioinformatics Analysis

The nucleotide sequence of goat *ELOVL1* cDNA and the deduced amino acid sequence were determined using BLAST [29]. Theoretical molecular weights of the deduced polypeptides and isoelectric points were predicted by calculating the isoelectric point [30]. Subcellular localization of ELOVL1 was predicted using PSORT [31]. Based on the amino acid sequence of ELOVL1, the functional domains were predicted using SMART [32] and the Pfam database [33]. Protein prosite patterns were identified using Psite [34]. Different phylogenetic trees were constructed from ELOVL1 DNA sequences using the MEGA4.1 software with the neighbor-joining method.

### 4.7. ELISA

Cells were collected and lysed in PBS by repeated freeze-thaw cycles, centrifuged to remove cellular debris, and assayed immediately or stored at −80 °C until analysis. ELOVL1 were measured per the ELISA kit manufacturer’s instructions (Catalog No. SEJ169Hu; *Cloud-Clone Corp*. Houston, TX, USA), and the absorbance was read at 450 and 570 nm on a Varioskan™ Flash Multimode Reader (Thermo Fisher Scientific, Pittsburgh, PA, USA).

### 4.8. Gas Chromatography and Mass Spectrum

Cells were treated with 50 nM rapamycin for 6 h, and then were collected and dissolved in 500 μL lysis buffer. Total lipids extraction with trichloromethane/methanol (*v*/*v* = 2:1) in an PBS-washed ampoule for GC-MS. After the transesterification with hydrochloric acid/methanol (*v*/*v* = 1:20) at 85 °C for 1 h, fatty acid methyl esters (FAME) were extracted with n-hexane at room temperature for 1 h and washed twice with water in preparation then separated in a gas chromatography-mass spectrum (Thermo TRACE 1310 GC-ISQ QD MS, Thermo Fisher Scientific Inc., 81 Wyman Street, Waltham, MA, USA, 02451) using a fused-silica capillary column with medium polarity (DB-5MS, 30 m × 0.25 mm × 0.25 μm, Agilent Technologies, Santa Clara, CA, USA). The initial capillary column chromatography program was set to column temperature 80 °C for 1 min, with ramping of 10 °C/min up to 200 °C, 5 °C/min up to 250 °C, 2 °C/min up to 270 °C, and a hold for 3 min. The injector temperature was 290 °C, the carrier gas flow rate was 1.2 mL/min, and the sample was 1 μL. The MS condition was set to solvent delay for 5 min, EI 70 eV, full scan mode, the scan quality in the range of 30~400 amu. FAME analytical standards obtained from Sigma-Aldrich. Nonadecanoic acid methyl ester is managed as an internal standard for quantitative analysis of fatty acids.

### 4.9. Western Blot and Antibodies

Inner Mongolia Cashmere GFbs were treated with rapamycin and washed 2 times with ice-cold PBS (pH 7.4). Cells were lysed in lysis buffer that contained 25 mM Tris-HCl (pH 7.6), 150 mM NaCl, 1% Nonidet P-40, 1% sodium deoxycholate, 0.1% SDS, protease inhibitor mixture and phosphatase inhibitors (Sigma, Chemicals) then placed on ice for 10 min. Next, the cells were harvested by scraping and centrifuged at 4 °C for 10 min at 13,000 rpm. Cells were lysed and the concentrations of protein lysates were measured using the Bio-Rad protein determination method (Bio-Rad Laboratories, Inc., Hercules, CA, USA).

Equal amounts of cell lysates were separated by SDS-PAGE on 10% polyacrylamide gels and transferred to PVDF membranes, which were then immunoblotted with the designated primary antibodies. The membranes were treated with horseradish peroxidase-conjugated goat anti-rabbit IgG or goat anti-mouse IgG (GE Healthcare, Little Chalfont, Buckinghamshire, UK) and detected using ECL (Thermo Fisher Scientific, Pittsburgh, PA, USA) by exposure to X-ray film. The resolved bands were quantified using Gel-Pro Analyzer 4.0.

Antibody to p70S6K (sc-230) was purchased from Santa Cruz (Dallas, TX, USA). Antibodies to ELOVL1 (ab74941) and phospho-p70S6K (Thr 389) (ab126818) were purchased from Abcam plc. (Cambridge, UK). Anti-β-actin (A5441) was obtained from Sigma Chemical (St. Louis, MO, USA).

### 4.10. Statistical Analysis

Descriptive statistics were generated for all quantitative data, which were expressed as mean ± SD. Each assay was performed in triplicate.

## 5. Conclusions

In this study, we cloned the cDNA that encodes Cashmere goat (*Capra hircus*) ELOVL1 (GenBank Accession number KF549985) and examined its expression in 10 tissues. *ELOVL1* cDNA is 840 bp, encoding a deduced protein of 279 amino acids, and *ELOVL1* mRNA is widely expressed in a variety of tissues. Inhibition of mTORC1 by rapamycin downregulates *ELOVL1* expression and fatty acid synthesis in Cashmere goat fetal fibroblasts. *ELOVL1* expression is regulated by mTORC1, and mTORC1 has significant function in fatty acid synthesis in Cashmere goat.

## Abbreviations

ELOVL1: elongation of very-long-chain fatty acids 1
DHA: docosahexaenoic acid
EPA: eicosapentaenoic acid
ELO: yeast elongase
GFb cells: goat fetal fibroblasts
mTOR: mammalian target of rapamycin
PPAR: peroxisome proliferator-activated receptor
and SREBP1: sterol regulatory element-binding protein 1
